# Viscoelasticity of articular cartilage: Analysing the effect of induced stress and the restraint of bone in a dynamic environment

**DOI:** 10.1016/j.jmbbm.2017.07.040

**Published:** 2017-11

**Authors:** Bernard M. Lawless, Hamid Sadeghi, Duncan K. Temple, Hemeth Dhaliwal, Daniel M. Espino, David W.L. Hukins

**Affiliations:** Dept. of Mechanical Engineering, University of Birmingham, Birmingham B15 2TT, UK

**Keywords:** Articular cartilage, Frequency, Loss, Modulus, Storage, Viscoelasticity

## Abstract

The aim of this study was to determine the effect of the induced stress and restraint provided by the underlying bone on the frequency-dependent storage and loss stiffness (for bone restraint) or modulus (for induced stress) of articular cartilage, which characterise its viscoelasticity. Dynamic mechanical analysis has been used to determine the frequency-dependent viscoelastic properties of bovine femoral and humeral head articular cartilage. A sinusoidal load was applied to the specimens and out-of-phase displacement response was measured to determine the phase angle, the storage and loss stiffness or modulus. As induced stress increased, the storage modulus significantly increased (*p* < 0.05). The phase angle decreased significantly (*p* < 0.05) as the induced stress increased; reducing from 13.1° to 3.5°. The median storage stiffness ranged from 548 N/mm to 707 N/mm for cartilage tested on-bone and 544 N/mm to 732 N/mm for cartilage tested off-bone. On-bone articular cartilage loss stiffness was frequency independent (*p* > 0.05); however, off-bone, articular cartilage loss stiffness demonstrated a logarithmic frequency-dependency (*p* < 0.05). In conclusion, the frequency-dependent trends of storage and loss moduli of articular cartilage are dependent on the induced stress, while the restraint provided by the underlying bone removes the frequency-dependency of the loss stiffness.

## 1 Introduction

Articular cartilage is a load bearing material found at the articulating ends of bones within joints of the body. Smooth joint motion is a result of the low friction at joints of the body, aided by a surface roughness of ~100 nm for articular cartilage (Ghosh et al., 2013). Osteoarthritis (OA) includes the degeneration of cartilage, leading to poor joint motion which is typically painful (Felson et al., 2000). Rapid heel-strike rise times, during gait, have been implicated in the onset of OA (Radin et al., 1991, Radin et al., 1986). These rapid heel-strike rise times were as low as 5–25 ms for the subset of the population potentially predisposed to OA (Radin et al., 1991, Radin et al., 1986). This is in contrast to estimated typical rise times of around 100–150 ms for otherwise healthy gait during walking (Fulcher et al., 2009). This rate of loading is important to the mechanical behaviour of cartilage, because its mechanical properties are rate dependent (Shepherd and Seedhom, 1997): cartilage is viscoelastic (Fulcher et al., 2009, Temple et al., 2016).

Viscoelastic materials can be characterised in terms of a storage, *E’*, and a loss, *E’’*, modulus (Hukins et al., 1999) while a viscoelastic structure can be characterised in terms of a storage, *k’*, and a loss, *k’’*, stiffness (Lawless et al., 2016). *E’* characterises the ability of the material to store energy for subsequent elastic recoil; whereas, *E’’* characterises the ability of the material to dissipate energy (Menard, 2008). The viscoelastic properties of cartilage have been characterised over frequencies ranging from typical gait frequencies (≥ 1 Hz) and up to frequencies representative of rapid heel-strike rise times (90 Hz) (Fulcher et al., 2009). The implication was that cartilage, on-bone, undergoes a glass transition at around 10–20 Hz, with a frequency-independent loss modulus but a storage modulus which increases with frequency. Subsequently, Sadeghi et al. (2015b) determined that frequency, independent of load, was significantly correlated to increased failure of articular cartilage. The mechanism proposed, consistent with the hypothesis provided by Fulcher et al. (2009), was that at higher frequencies, the storage modulus increases but the loss modulus remains constant. Thus, the ability of the tissue to store energy is greater at higher frequencies. This increased energy, past a certain point, predisposes the tissue to undergo failure; thereby, dissipating energy within the tissue. Frequencies above a proposed glass transition (Sadeghi et al., 2017a, Sadeghi et al., 2017b; Sadeghi et al., 2015b) appear to be of particular concern regarding failure.

Induced stresses in articular cartilage have been estimated to range from 1 to 6 MPa for moderate activities, such as walking (Ahmed and Burke, 1983, Brown and Shaw, 1983, Hodge et al., 1989) with peak stresses estimated to reach up to 10.7 MPa, for stair-climbing, and 18 MPa for rising from a chair (Hodge et al., 1989). This is in comparison to induced stresses of around 1–1.7 MPa estimated for hip and knee joints during ‘ambulatory’ activities, i.e. walking (Yao and Seedhom, 1993). The material properties of cartilage have previously been found to be strain-dependent (Barker and Seedhom, 2001). However, the relationship was not linear, instead resembling a U-shaped relationship. Different stress levels imply different strain, and potentially different mechanical response to loading (Barker and Seedhom, 2001). The relevance, though, of stress to the dynamic viscoelasticity of cartilage is currently unknown.

The juxtaposition of cartilage and bone will mean that a change in one will lead to a change on stress generated with the other (Dar and Aspden, 2003); hence, the relevance of understanding the interactions between articular cartilage and bone. The underlying subchondral bone to which cartilage is attached, has a restrictive effect (Aspden, 1990) on cartilage and prevents lateral displacement at the base of the tissue (Burgin and Aspden, 2008). For example, it has been suggested that the underlying bone would attenuate the increased energy dissipation with loading velocity observed off bone (Edelsten et al., 2010). The extrapolated implication being that cartilage on- and off-bone have different frequency-dependent loss moduli. This inference appears to be consistent with the finding that cartilage off-bone has a frequency-dependent loss modulus (Aspden, 1991, Temple et al., 2016), as opposed to a frequency-independent when on-bone. However, differences between testing procedures could make this inference invalid. For example, testing of cartilage samples in air as opposed to within a hydrating solution (e.g. Ringer’s solution); since hydration alters the viscoelastic properties (Pearson and Espino, 2013) and predisposition to failure of articular cartilage (Fick and Espino, 2012).

The aim of this study was to determine the effect of the induced stress and restraint provided by the underlying bone on the frequency-dependent viscoelastic properties of articular cartilage. Some tests were performed on cartilage in a hydrating fluid (Ringer’s solution) and others in air, in order to understand the limitations of comparing published studies performed under these different conditions. Except for bone restraint, viscoelasticity has been analysed in terms of *E’* and *E’’*. Bone restraint, has been analysed in terms of *k’* and *k’’*, since the combination of cartilage and bone is a structure and not a material.

## 2 Materials and methods

### 2.1 Specimens

Three bovine femoral heads and eight bovine humeral heads, of approximately between 18 and 30 months old, were obtained from a supplier (Dissect Supplies, Birmingham, UK); bovine cartilage is a suitable model for the dynamic viscoelasticity of human cartilage (Temple et al., 2016). Specimens were wrapped in tissue paper, and saturated in Ringer’s solution, on arrival in the laboratory. Specimens were then stored in a freezer at −40 °C. Specimens were thawed for 12 h before testing. Freeze-thaw treatment does not alter the dynamic mechanical properties of articular cartilage (Szarko et al., 2010). Large scale damage of the cartilage on joints was not evident. However, India Ink (Loxley Art Materials, Sheffield, UK) was used to ensure that only intact surfaces were used for testing (Aspden, 2011, Meachim, 1972) because surface cracks alter the mechanical properties of articular cartilage (Burgin and Aspden, 2008).

Sixteen cylindrical test specimens (see Table 1) were obtained using a cork borer with a medical scalpel used to isolate the cartilage from the subchondral bone (Burgin and Aspden, 2008, Edelsten et al., 2010, Lewis et al., 1998, Temple et al., 2016). The specimens were 5.2 mm in diameter, but varied in thickness (see Table 1).

**Table 1:** Cartilage specimens used for testing protocols. Thickness of the femoral head specimens used in the testing of articular cartilage in air / in Ringer’s solution and thickness of the humeral head specimens used to analyse stress dependency of the viscoelastic properties; the diameter of these specimens was 5.2 mm. Humeral head core specimens used to analyse the effect of the restraint of bone on the viscoelastic properties; the diameter of the core specimens was 4.1 mm.

In Air / In Ringer’s			Stress dependency			Restraint of Bone	
Joint ID	Specimen ID	Thickness (mm)	Joint ID	Specimen	Thickness (mm)	Joint ID	Specimen
Femoral Head 1	Specimen 1	1.18	Humeral Head 1	Specimen 1	0.77	Humeral Head 1	Core Specimen 1
Femoral Head 3	Specimen 2	1.28	Humeral Head 2	Specimen 2	1.02	Humeral Head 2	Core Specimen 2
Femoral Head 3	Specimen 3	1.15	Humeral Head 3	Specimen 3	1.10	Humeral Head 3	Core Specimen 3
Femoral Head 2	Specimen 4	0.88	Humeral Head 4	Specimen 4	1.04	Humeral Head 4	Core Specimen 4
Femoral Head 1	Specimen 5	1.35	Humeral Head 5	Specimen 5	0.66	Humeral Head 5	Core Specimen 5
Femoral Head 3	Specimen 6	1.02	Humeral Head 6	Specimen 6	1.23	Humeral Head 6	Core Specimen 6
Femoral Head 2	Specimen 7	1.05	Humeral Head 7	Specimen 7	0.95	Humeral Head 7	Core Specimen 7
Femoral Head 2	Specimen 8	1.03	Humeral Head 8	Specimen 8	1.22	Humeral Head 8	Core Specimen 8
	Mean ± Std. Dev.	1.12 ± 0.15		Mean ± Std. Dev.	1.00 ± 0.20		

### 2.2 DMA frequency sweep

A Bose ElectroForce 3200 testing machine running WinTest 4.1 Dynamic Mechanical Analysis (DMA) software (Bose Corporation, Minnesota, USA; now, TA Instruments, New Castle, DE, USA) was used to quantify the viscoelastic properties. This approach has been used to characterise the viscoelastic properties of natural tissues (Barnes et al., 2016, Barnes et al., 2015, Burton et al., 2017, Fulcher et al., 2009, Temple et al., 2016) and orthopaedic implants (Lawless et al., 2017, Lawless et al., 2016). Each test specimen underwent a frequency sweep (1, 8, 10, 12, 29, 49, 71, and 88 Hz), following preloading at 25 and 50 Hz (1500 and 3000 cycles, respectively, with a 60 s rest period). For each frequency, the DMA software calculated a storage (*k’*) and loss (*k’’*) stiffness as shown in Eqs. (1), (2), (3); where k*, F* and d* are the magnitude of the complex stiffness, the magnitude of the force (from the Fast Fourier Transform, FFT, of the sinusoidal force wave) and the magnitude of the displacement (from the FFT of the sinusoidal displacement response wave), respectively. Further details can be found elsewhere (Lawless et al., 2016).

(1) $$k^{*}=\frac{F^{*}}{d^{*}}$$

(2) $$k^{\prime }=k^{*}\cos\delta$$

(3) $$k^{\prime \prime }=k^{*}\sin\delta$$

The angle *δ* is the phase difference between the applied compressive force and the displacement.

A 20 mm diameter compression plate was used to compress articular cartilage specimens. This DMA frequency sweep was used for three different testing procedures described in Section 2.3.

### 2.3 Testing protocols

The DMA frequency-sweep was applied under three distinct testing protocols which focused on test specimens: (1) in air and in Ringer’s solution; (2) loaded under different levels of sinusoidal loading to vary the induced stress; and (3) on- and off-bone.

For testing protocol-1, 8 test specimens (all from the femoral head; see Table 1) were tested following the DMA procedure (Section 2.2) in air or in Ringer’s solution. To enable a paired comparison, each individual test specimen was tested under both conditions with half the test specimens tested first in air and the other half first in Ringer’s solution. Between tests, each specimen was allowed to rest/recover whilst saturated in Ringer’s solution for 30 min; this ensured cartilage returned to a hydrated state prior to the subsequent test, consistent with literature (Barker and Seedhom, 2001, Park et al., 2004). A sinusoidally compressive force was applied between 16 and 36 N (Fulcher et al., 2009, Temple et al., 2016). Peak loading induced maximal stresses of 1.7 MPa, estimated physiological for lower limb cartilage during walking (Yao and Seedhom, 1993).

For testing protocol-2, 8 test specimens, from the humeral head, were tested in air following the DMA procedure (Section 2.2) with a variety of three different sinusoidal loading ranges: (a) 2–22 N; (b) 16–36 N and (c) 65–85 N. This induced three different ranges of dynamic stress (Table 2). To enable paired comparisons, each specimen was tested under the three loading ranges with the order of testing varied with Excel Random Function (Redmond, Washington, USA).

**Table 2:** Loading conditions and induced stress ranges used to determine the stress dependency of the viscoelastic properties of cartilage.

Stress dependency			
Induced Stress Range	Induced Stress Range (MPa)	Applied Load Range (N)	Dynamic Amplitude (N)
Low	0.09 – 1.04	2 – 22	20
Walking	0.75 – 1.70	16 – 36	20
High	3.06 – 4.00	65 – 85	20

For testing protocol-3, 8 test specimens were obtained from humeral heads and tested on-bone and then off-bone. These samples were not cut using a cork borer (discussed above) but by using a hollow drill-head attached to a drill (Burgin and Aspden, 2008). Cylindrical cartilage on bone specimens were obtained, 4.1 mm in diameter. These specimens underwent the DMA procedure outlined, above in the Section 2.2, first on-bone and subsequently after using a medical scalpel to isolate the cartilage from the bone. For both cartilage specimens on- and off-bone, a sinusoidally compressive force was applied between 10 and 24 N. This loading range induced a maximal stress of 1.8 MPa, comparable to the estimated cartilage walking peak stress of 1.7 MPa (Yao and Seedhom, 1993).

Following testing for testing protocols 1 and 2, cartilage thickness was measured for each test specimen (Table 1) (Shepherd and Seedhom, 1999). Briefly, a sharp needle was pushed through the layer of articular cartilage and up to the underlying plate (using the testing machine). The specimen diameter (*D* = 5.2 mm) and thickness (*t*; see Table 1) were then used to calculate a shape factor (*S*; Eq. (4)) from which the magnitude of the complex modulus (E*), the storage modulus (*E’*) and loss modulus (*E’’*) were determined using Eqs. ((5), (6), (7), respectively; further details are available elsewhere (Espino et al., 2014, Fulcher et al., 2009).

(4) $$S=\frac{\pi D^{2}}{4t}$$

(5) $$E^{*}=\frac{k^{*}}{S}$$

(6) $$E^{\prime }=\frac{k^{\prime }}{S}$$

(7) $$E^{\prime \prime }=\frac{k^{\prime \prime }}{S}$$

Storage (*k’*) and loss (*k’’*) stiffness were used in test protocol-3 as measuring the thickness was not feasible. To understand the effect of stress to the potential failure of cartilage, the ratio of storage modulus to loss modulus (*E’/E’’*) was calculated for every frequency for test protocol-2. For protocol-3, the ratio of storage stiffness to loss stiffness (*k’/k’’*) was calculated to understand how the restraint of bone affects the potential failure of cartilage.

### 2.4 Data analysis

All statistical comparisons were performed using SigmaPlot 13.0 (SYSTAT, San Jose, CA, USA). For test protocol-3, the logarithmic frequency-dependent behaviour of *k’* and *k’’* were described according to Eqs. (8), (9), respectively, where *A, A*_*L*_, *B* and *B*_*L*_ were determined to give the least-squares best fit.

(8) $$k^{\prime }=A\log_{e}\left(f\right)\;+\;B$$

(9) $$k^{\prime \prime }=A_{L}\log_{e}\left(f\right)+B_{L}$$

For test protocol-1 and -2, *E’* and *E’’* were described according to Eqs. (10), (11), respectively, where *C*, *C*_*L*_, *D* and *D*_*L*_ were determined to give the least-squares best fit.

(10) $$E^{\prime }=C\log_{e}\left(f\right)\;+\;D$$

(11) $$E^{\prime \prime }=C_{L}\log_{e}\left(f\right)+D_{L}$$

Wilcoxon signed rank tests were used to compare the *E’* and *E’’* of cartilage when tested in air versus in Ringer’s solution (i.e. test protocol-1). Wilcoxon signed rank tests were also used to compare *k’*, *k’’ and k’/k’’* of cartilage on- and off-bone (i.e. test protocol-3). A Friedman repeated measures analysis of variance (ANOVA) on ranks was performed to evaluate the differences between cartilage specimens tested at different stress ranges (i.e. test protocol-2). If the Friedman test showed a significant difference between the groups (*p* < 0.05), a Student-Newman-Keuls (SNK) multiple comparison test was used to determine the differences between the groups in relation to *E’*, *E’’*, *E’/E’’* and *δ* (phase angle between the applied force and material deformation). The results of all statistical tests with a *p* < 0.05 were considered significant.

## 3 Results

### 3.1 Test medium

**Fig. 1:**
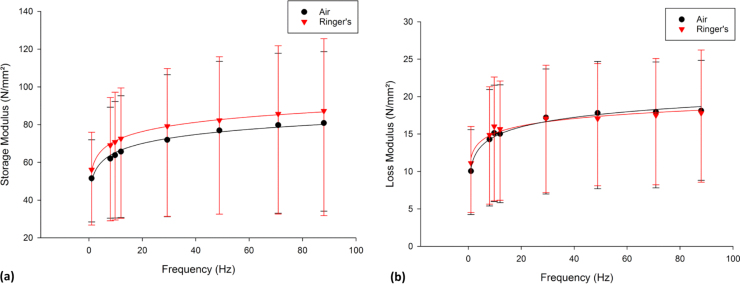
Frequency-dependent viscoelastic properties, (a) storage and (b) loss modulus (N/mm^2^), of articular cartilage tested in air and in Ringer’s solution (median ± 95% confidence intervals, [n = 3] with natural logarithmic regression trendlines). In total 8 specimens from 3 femoral heads were tested.

The testing of articular cartilage off-bone, in either air or Ringer’s solution, did not alter the general logarithmic trend (*p* < 0.05) of either *E’* (Eq. (10)) or *E’’* (Eq. (11)) in relation to frequency (Fig. 1). *E’* was not significantly different (*p* > 0.05), for cartilage tested in air (range 52–81 MPa) and in Ringer’s solution (range 56–87 MPa), for any frequency tested. Likewise, *E’’* was not significantly different (*p* > 0.05), for cartilage tested in air (range 10–18 MPa) and in Ringer’s solution (range 11–18 MPa), for any frequency tested.

### 3.2 Stress dependency of viscoelasticity

**Fig. 2:**
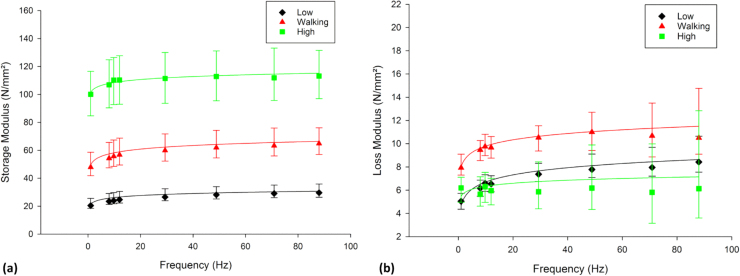
Frequency-dependent viscoelastic properties, (a) storage and (b) loss modulus (N/mm^2^), of articular cartilage tested at low, normal walking and high stress ranges (median ± 95% confidence intervals, [n = 8] with natural logarithmic regression trendlines). In total 8 specimens from 8 humeral heads were tested in air.

**Fig. 3:**
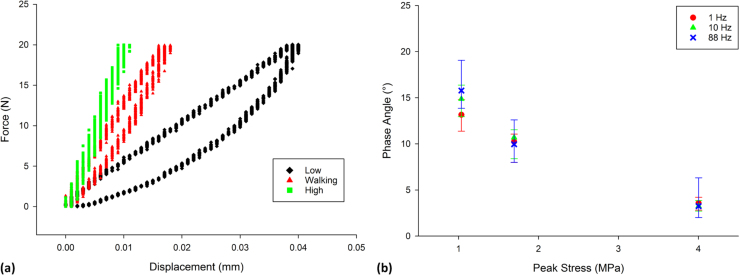
Effect of loading range on viscoelastic behaviour. (a) Force-displacement hysteresis loops, of Specimen 4, at low, walking and high stress (frequency at 10 Hz); the hysteresis loops illustrated here are similar to other specimens as well as different frequencies. (b) changes in phase angle, *δ*, in relation to the induced stress (median ± 95% confidence intervals; n = 8).

The viscoelastic response of articular cartilage varied with the induced stress (Fig. 2 and Fig. 3a). Increasing the induced sinusoidal stress from low stress to high stress led to a significant increase (*p* < 0.05; Table 3) of *E’* by 3.8 (88 Hz) to 4.9 (1 Hz) times (Fig. 2). The logarithmic trend of the frequency-dependency of *E’* (*p* < 0.05; Table 4) did not change at low, walking or high stress; it was off-set between the groups. *C* did not vary (2.1–3.8 MPa); however, *D* increased from 19.6 to 102 MPa (Table 4) as the induced stress increased.

**Table 3:** Student-Newman-Keuls multiple comparison test results between viscoelastic properties calculated from the different induced stress ranges; Low, walking and High. *p* < 0.05 indicates that the comparison were significantly different and the *p*-values did not vary with varying frequency (1–88 Hz).

Frequency	Storage Modulus (N/mm^2^) *E’*			Loss Modulus (N/mm^2^) *E’’*		
Hz	High/Low	High/walking	walking/Low	High/Low	High/walking	walking/Low
1 to 88	*p* < 0.05	*p* < 0.05	*p* < 0.05	*p* > 0.05	*p* < 0.05	*p* < 0.05

**Table 4:** Storage stiffness (*k’*), loss stiffness (*k’’*), storage modulus (*E’*) and loss modulus (*E’’*) regression analyses. Stiffness coefficients (*A* and *B*) are in N/mm while moduli coefficients (*C* and *D*) are in MPa. Coefficients for the median trends are provided. *p* < 0.05 indicates that the logarithmic regression analysis was significant.

*Test Protocol*	*Test*	*Storage Property*	*A*	*B*	*C*	*D*	*r*^*2*^	*p value*
1	Air	*E'*	–	–	6.82	49.6	0.983	< 0.05
1	Ringer’s Solution	*E'*	–	–	7.03	55.3	0.997	< 0.05
2	Low Stress Range	*E'*	–	–	2.14	19.6	0.974	< 0.05
2	Walking Stress Range	*E'*	–	–	3.76	47.3	0.992	< 0.05
2	High Stress Range	*E'*	–	–	2.77	102	0.887	< 0.05
3	On Bone	*k'*	35.4	544	–	–	0.996	< 0.05
3	Off Bone	*k'*	43.3	533	–	–	0.987	< 0.05
*Test Protocol*	*Test*	*Loss Property*	*A*_*L*_	*B*_*L*_	*C*_*L*_	*D*_*L*_	*r*^*2*^	*p value*
1	Air	*E''*	–	–	1.84	10.5	0.978	< 0.05
1	Ringer’s Solution	*E''*	–	–	1.42	11.8	0.936	< 0.05
2	Low Stress Range	*E''*	–	–	0.74	4.87	0.979	< 0.05
2	Walking Stress Range	*E''*	–	–	0.64	8.12	0.919	< 0.05
2	High Stress Range	*E''*	–	–	−0.03	6.09	0.027	0.695
3	On Bone	*k''*	0.36	55.7	–	–	0.127	0.387
3	Off Bone	*k''*	2.82	69.0	–	–	0.774	< 0.05

*E’’* varied with the induced stress (*p* < 0.05), with significantly higher values of *E’’* for walking stress than low and high stress. There was no significant difference in *E’’* between low and high stress (*p* > 0.05; Table 3). The frequency-dependent *E’’* was logarithmic for low and walking stress ranges tested (*p* < 0.05; Table 4), however, *E’’* did not follow a frequency-dependent logarithmic trend for the high stress range (*p* > 0.05; Table 4). *C*_*L*_ did not vary between the low stress range (0.74 MPa) and walking stress range (0.64 MPa). *D*_*L*_ increased from 4.9 MPa (low stress) to 8.1 MPa (walking stress; Table 4).

**Fig. 4:**
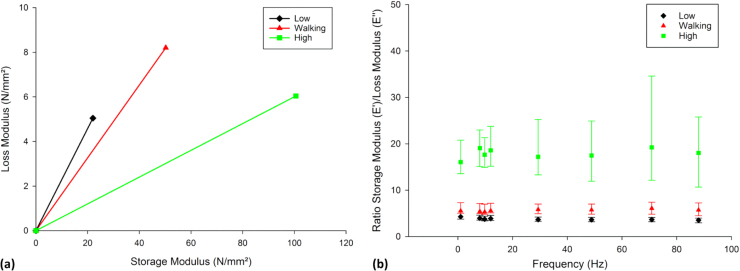
Effect of stress on storage and loss moduli. (a) Argand diagram showing the storage and loss moduli of articular cartilage tested at low, walking and high stress ranges. The plotted lines demonstrate the mean magnitude of the complex modulus (E*) of the low, walking and high stress ranges at 1 Hz; the Argand diagram illustrated here is similar to other specimens as well as different frequencies. (b) Ratio of storage modulus/loss modulus of articular cartilage tested between at low, walking and high stress ranges (median ± 95% confidence intervals; n = 8). The raw data used in Fig. 2 is also used in Fig. 4b, however, Fig. 4b is not derived directly from Fig. 2.

Hysteresis loops were larger for the lowest levels of induced stress (Fig. 3a); the reduction of the area within the centre of the hysteresis loop demonstrates that as the induced stress increases, cartilage dissipates less energy (i.e. an increasingly elastic response). This corresponded to a much larger phase angle, between stress and strain, at such stresses, which decreased significantly with increased level of median induced stress (*p* < 0.05); for example, at 1 Hz *δ* decreased from 13.1° to 3.5° at 1 Hz with increasing stress level (Fig. 3b). The decrease in phase angle, as the induced stress increases, also highlights an increasingly elastic response. This was accompanied by an increase in E* from 23 MPa at the lowest level of induced stress to 51 MPa at walking induced stresses, increasing to 101 MPa at high induced stresses (Fig. 4a).

Walking stresses *E’/E’’* was significantly different (*p* < 0.05) between the three induced stress groups for every frequency, increasing with induced stress. At the higher stress range, *E’:E’’* was 2.90–3.52 times greater than *E’/E’’* for the walking stress range (Fig. 4b). For the walking stress range, *E’/E’’* was 1.29–1.66 times greater than the low stress range.

### 3.3 Restraint of underlying bone

**Fig. 5:**
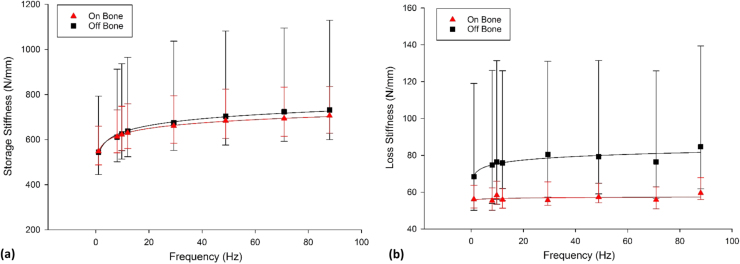
Frequency-dependent viscoelastic properties, (a) storage and (b) loss stiffness (N/mm), of articular cartilage on bone and off bone (median ± 95% confidence intervals, [n = 8] with logarithmic regression trendlines). In total 8 specimens from 8 humeral heads were tested.

The median *k’* ranged from 548 N/mm to 706 N/mm for on-bone and 544 N/mm to 732 N/mm for off-bone (Fig. 5) and *k’* was logarithmically frequency-dependent for both on- and off-bone (*p* < 0.05; Eq. (8); Table 4). For all frequencies tested, *k’* was not significantly different when on- and off- bone (*p* > 0.05; Wilcoxon signed rank test).

The frequency-dependency of *k’’* varied for articular cartilage when on and off-bone. On-bone articular cartilage was frequency independent (*p* > 0.05; Table 4). However, for off-bone, articular cartilage *k’’* demonstrated a frequency-dependency. Regression analysis demonstrated that this frequency-dependency could be empirically described using Eq. (9) (p < 0.05; Table 4). *k’’*, off-bone, was significantly greater than on-bone (*p* < 0.05; Wilcoxon signed rank test) for all frequencies except for 1 Hz (*p* = 0.055). The on-bone *k’/k’’* ratio was significantly greater than cartilage off-bone for all frequencies tested (*p* < 0.05).

**Fig. 6:**
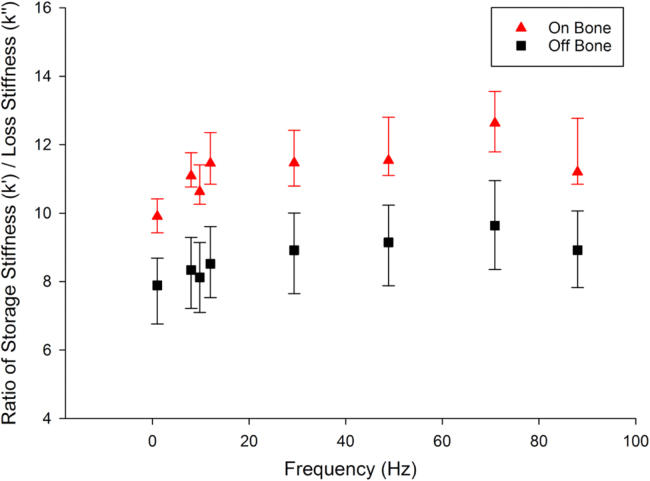
Ratio of storage stiffness/loss stiffness of articular cartilage on-bone and off-bone (median ± 95% confidence intervals). In total 8 specimens from 8 humeral heads were tested.

The ratio of *k’/k’’* for articular cartilage on-bone ranged between 9.91 (1 Hz) to 12.63 (71 Hz) while off-bone it ranged between 6.65 (1 Hz) to 10.23 (12 Hz). The ratio of *k’/k’’* for on-bone articular cartilage was 1.26–1.34 times greater than *k’/k’’* off-bone (Fig. 6); these differences were significant for every frequency (*p* < 0.05).

## 4 Discussion

### 4.1 Outline

This study has, firstly, assessed the effect of induced stress on the frequency-dependent viscoelastic properties of articular cartilage. The storage modulus increased with induced stress. However, the loss modulus peaked at induced stresses associated with walking. Changes in storage and loss moduli were reflected in significant decreases in the stress-strain phase lag, and an increased magnitude for the complex modulus (Eq. (5)), with increased mean level of induced stress. Secondly, the restraint provided by bone was found to alter the loss, but not the storage, stiffness dependency at all loading frequencies above 1 Hz. Off-bone, cartilage was found to have a frequency-dependent loss stiffness, and although the loss stiffness demonstrated increased variability it increased off-bone. Finally, the frequency-dependent viscoelasticity of cartilage specimens was not altered by the being exposed to air or surrounded in Ringer’s solution; generic trends may be compared directly across studies which have used comparable stress and on-/off-bone test protocols. It should be noted, though, that during the DMA frequency-sweeps in air (which lasted ~10 min), only the circumference along the length of the cylindrical sample was exposed to air.

In extrapolating the results from this study, it should be noted that bovine cartilage was used as a model for human cartilage. Human cartilage, however, is around 0.3–0.5 mm thicker than bovine cartilage (Taylor et al., 2011, Temple et al., 2016). Bovine cartilage is a recognised dynamic model for human cartilage, but the storage and loss moduli of human cartilage are around half the value of the respective measurements for bovine cartilage (Temple et al., 2016). The frequency-dependent trends over a physiological range of induced stresses, though, are analogous. Therefore, with the proviso of a multiple of 2, the bovine model can approximate the dynamic behaviour of human articular cartilage (Temple et al., 2016).

### 4.2 Boundary conditions, energy and failure

In this current study, the storage modulus increased significantly with the magnitude of induced stress. Further, the storage modulus of cartilage across all levels of induced stress was frequency-dependent. At the highest levels of induced stress and at the higher frequencies, the storage modulus was of the order of 0.1 GPa. These values are easily an order of magnitude above that reported elsewhere for elastic moduli (Shepherd and Seedhom, 1999), and not much lower than the moduli of the underlying bone (Burgin and Aspden, 2008). However, our finding is consistent with studies which have measured: increased dynamic moduli with increased induced stress derived from the slope of the stress strain curves (Park et al., 2004); and, the moduli of articular cartilage to approximate that of the underlying cancellous bone during impact loading (Burgin and Aspden, 2008). All of which is consistent with the proposition that cartilage undergoes a glass transition, so that at higher rates of loading, and/or higher frequencies, it becomes more rigid (Fulcher et al., 2009) but also more prone to failure (Sadeghi et al., 2017a, Sadeghi et al., 2017b; Sadeghi et al., 2015b).

Unlike the storage modulus, the loss modulus did not simply increase with induced stress. Instead, the loss modulus was greatest at induced stresses associated with walking, decreasing with both lower and higher induced stress. This trend shares some parallels with the compressive modulus of cartilage displaying a second-order polynomial strain-dependency (Barker and Seedhom, 2001). Barker and Seedhom (2001) hypothesised that cartilage adapts its matrix constituents to be least susceptible to damage by minimising total strain, through a balance of viscous and elastic strain. Our finding of a loss modulus which peaks during stresses induced during walking appears consistent with their hypothesis. It seems feasible that the loss modulus increases towards stresses associated with walking (i.e. to match energy dissipation with increased loading), but that as compression becomes excessive the ability of collagen and its surrounding gel phase to interact and dissipate energy may be impaired; a limited ability to naturally dissipate energy may, thus, result in damage. This concept is consistent with the suggestion by Burgin and Aspden (2008) that failure inducing impacts were less elastic with increased impact energy due to dissipative effects of internal tissue damage and crack formation.

The loss stiffness of articular cartilage increased with frequency when off-bone, but remained frequency-independent when on-bone: an anticipated finding. Fulcher et al. (2009) found the loss modulus of on-bone cartilage to be frequency-independent; whereas, cartilage off-bone has a frequency-dependent loss modulus (Aspden, 1991, Temple et al., 2016). Edelsten et al. (2010) had previously argued that off-bone cartilage would lead to an increase in loss modulus, which would differ from on-bone cartilage. This was based on measurements off-bone, and a previous analytical study on the restraining effect of surrounding structures (Aspden, 1990). The subchondral bone restrains the lateral expansion of the tissue in the deep zone (Park et al., 2004); thus, its removal may enable cartilage to deform laterally more freely leading to increased hysteresis during a loading cycle. It should be noted that in our current study, viscoelastic properties of on- and off-bone cartilage were compared by structural stiffness rather than modulus. This was done to remove any bias in comparison which might ensue from assumptions around the cartilage shape factor. However, as the shape factor used in calculations (Eqs. (5), (6), (7)) is ultimately a constant this does not alter frequency-dependent trends (i.e. a stiffness and modulus cannot be compared, but their frequency-dependency can).

There was no dependency of the storage stiffness on the presence/absence of underlying subchondral bone; unlike the loss stiffness. Therefore, on-bone cartilage was calculated to have a higher *storage/loss* ratio, around 1.3 times greater than off-bone. Thus, on-bone cartilage may be more predisposed to failure than off-bone cartilage. This is not surprising as energy from potentially damaging loading, off-bone, might be dissipated via increased hysteresis; however, on-bone cartilage might dissipate excess energy through the formation of cracks in the cartilage. Clearly, propensity to failure is multi-factorial, dependent on factors including: high induced stresses (which increased the *storage/loss* ratio by up to 3.5 when compared to walking induced stresses); hydration (Pearson and Espino, 2013); and frequency of loading (Sadeghi et al., 2017a, Sadeghi et al., 2017b; Sadeghi et al., 2015b). Further, while regions with differing matrix integrity across a joint (Bullough et al., 1985) may have a similar *storage/loss* ratio (Espino et al., 2014), a compromised matrix may well have a lower *storage/loss* failure threshold (i.e. less energy required for failure). This is likely related to the mechanism by which collagen interacts with the surrounding gel (Goh et al., 2007, Goh et al., 2005, Ng et al., 2010).

Alterations to collagen-gel interaction appear to lead to increased storage modulus with increased loading, but an altered loss modulus when bone restrains cartilage. Energy transfer mechanisms during plastic deformation of ground substance over collagen fibres/fibrils (Goh et al., 2007, Goh et al., 2005) or once collagen fibrils/fibres have exceeded a critical length (Goh et al., 2003, Hukins and Aspden, 1985) could both be implication in failure. There is also the potential for fibre-pullout (Goh et al., 2004) at the bone-cartilage interface, which may subsequently increase localised hysteresis near deeper cartilage layers. A hypothesised glass transition (Fulcher et al., 2009) appears consistent with an expectation of increased failure with increased frequency of loading, because of the increase in *storage/loss* ratio.

The importance of the internal swelling pressure of cartilage in resisting compressive stress has some important implications for the results described here. The gel surrounding the collagen fibrils is polyanionic and attracts water by the Donnan effect; this effect leads to cartilage having an internal swelling pressure or ‘turgor’ that enables it to withstand applied compression (Maroudas, 1976). In the resting tissue this pressure is balanced by tension in the collagen fibrils (Maroudas, 1976). The collagen fibrils in articular cartilage are oriented so that they are placed in tension by the internal pressure, leading to mechanical equilibrium of the tissue (Aspden and Hukins, 1981, Hukins et al., 1984). Increasing the compressive stress applied to the cartilage surface will then increase its stiffness, provided that the collagen network is not damaged, that little fluid is expressed by the tissue and that the viscosity of the tissue is not too great. It might be expected that removing the cartilage from the underlying bone would disrupt the collagen network and so affect the storage stiffness. However, the collagen fibrils in this region are oriented to prevent the swelling pressure from lifting the cartilage off the bone (Aspden and Hukins, 1981, Hukins et al., 1984). This effect of the swelling pressure is not likely to be important in a laboratory compression test, so removal of the mechanism for its prevention is unlikely to be important. It might be considered that, given the importance of tissue hydration for mechanical properties, that mechanical tests of articular cartilage should be performed in a hydrating fluid. However, this will only be true if fluid expression is an important factor in the mechanical response of cartilage in the time-scale of the tests. Evidence from the response of cartilage to impact loading and the published values of cartilage permeability, suggest that fluid flow and fluid expression may be less important than is commonly supposed (Burgin and Aspden, 2008, Edelsten et al., 2010).

### 4.3 Physiological stresses

In this study, stresses were induced in the range of 0.09–4 MPa. The range investigated incorporates low stress (~0.12 MPa) studies (Taylor et al., 2011), stresses estimated as physiological during walking (Espino et al., 2014, Fulcher et al., 2009, Park et al., 2004, Sadeghi et al., 2015a, Swann and Seedhom, 1993, Temple et al., 2016), and greater, but still physiological, stresses (1.7–3.4 MPa) (Zimmerman et al., 1988). Induced stresses associated with cartilage failure of above 4 MPa were avoided (Sadeghi et al., 2015b). The range investigated was lower than failure stresses associated with creep loading of around 8–10 MPa (Fick and Espino, 2012, Fick and Espino, 2011), or induced during traumatic loading of 10–40 MPa (Milentijevic and Torzilli, 2005) or 25–50 MPa (Jeffrey and Aspden, 2006). It is noted that at induced stresses in the range of 50 MPa, Jeffrey and Aspden (2006), calculated a ‘dynamic’ modulus (the maximum value of the differentiated stress-strain curve, often referred to as a tangent modulus) as 170 ± 21 MPa for bovine cartilage. This is higher than the values reported in this present study, in which the storage modulus did not exceed 114 MPa; however, higher induced stress would be expected to lead to a higher material rigidity, demonstrating consistency between the premise of the two studies.

Equilibrium and aggregate (an equilibrium modulus following the cessation of fluid flow through the tissue) moduli of less than 1 MPa reported (Athanasiou et al., 1991, Taylor et al., 2011) are orders of magnitude lower than the moduli reported in this current study, or impact studies (Jeffrey and Aspden, 2006). However, induced stresses during such creep (or stress relaxation) based studies are typically below those estimated as physiological during walking (1–1.7 MPa) for lower limb cartilage (Swann and Seedhom, 1993, Yao and Seedhom, 1993). Results from our current study have demonstrated that at stresses below the 1–1.7 MPa range, the storage modulus decreased significantly, with the force-displacement phase lag increasing up to 15° (and a much larger hysteresis loop than at walking stresses). Further, it has previously been demonstrated that low loading frequencies also exaggerate this phase lag (Park et al., 2004, Sadeghi et al., 2015a). Thus, if the effects of creep testing and low induced stresses are additive, cartilage may exhibit viscoelastic behaviour which is far removed from cartilage under walking conditions. The dissipative effects of cartilage will present as enhanced, evidenced by a phase angles of ≥15° and reduced magnitude of the complex moduli (or equivalent moduli). Therefore, it may appear to behave in more ‘viscous’ manner than is physiological. Cartilage might appear to be dominated by fluid exudation, while at physiological loading rates the matrix may better approximate an elastic solid (Burgin and Aspden, 2008; Edelsten et al., 2010).

## 5 Conclusion

The conclusions from the frequency-dependent viscoelastic properties of articular cartilage are that:

• articular cartilage is proportionally ‘more viscous’ at low stress and, therefore, not a representation of physical behaviour under a physiological stress range;
• at a high induced stress range, articular cartilage is ‘more elastic’ in response when compared to the walking stress range;
• off-bone articular cartilage has a greater ability to dissipate energy and its loss stiffness is frequency-dependent, while on-bone articular cartilage is frequency-independent;
• there is no significant difference in viscoelastic properties, in relation to frequency, of articular cartilage whether tested, for short tests (time < 10 min), in air or in Ringer’s solution.
